# Inadequate toothbrushing practice and associated factors among in-school adolescents in Malaysia: Findings from Global School Health Survey (GSHS) 2017

**DOI:** 10.1371/journal.pone.0317484

**Published:** 2025-01-31

**Authors:** Mohamad Fuad Mohamad Anuar, Nurulasmak Mohamed, S. Maria Awaluddin, Habibah Yacob

**Affiliations:** 1 Biostatistics and Repository Data Sector, Office of NIH Manager, National Institute of Health, Ministry of Health Malaysia, Shah Alam, Malaysia; 2 Oral Health Epidemiology and Research Unit, Oral Health Program, Ministry of Health Malaysia, Putrajaya, W.P. Putrajaya, Malaysia; 3 Institute for Public Health, National Institute of Health, Ministry of Health Malaysia, Shah Alam, Selangor, Malaysia; 4 Oral Health Regulations and Practice Division, Oral Health Program, Ministry of Health Malaysia, Putrajaya, W.P. Putrajaya, Malaysia; Universidade Federal de Pelotas, BRAZIL

## Abstract

**Introduction:**

Inadequate toothbrushing practice is define as brushing teeth less than two times per day. Inadequate toothbrushing during adolescence can lead to oral health problems and disease burden in adults. Moreover, inadequate practice can lead to low quality of life and inadequate self-esteem.

**Objective:**

This study aimed to determine the prevalence of inadequate toothbrushing practice among adolescents aged 13 to 17 years in Malaysia and its association with sociodemographic and other related risky lifestyles.

**Method:**

This study was part of a national cross-sectional study, Global School Health Survey 2017. 27,497 students were agreed to participate in this study, with response of 89.2%. A validated self-administered bilingual, comprised of topics related to sociodemographic as well as adolescent health and risky lifestyles; substance use (alcohol, drug, smoking cigarettes), eating patterns, hygiene (inclusive of oral and hand hygiene), mental health status, lack of peer and parental/guardian support, truancy, physical activity, and body mass index (BMI). Analysis was performed using IBM SPSS for Windows version 26.0 involving complex sampling analysis and logistic regression.

**Results:**

A total of 12.7% (95% CI: 11.8–13.6) of in-school adolescents had inadequate toothbrushing practices. Higher prevalence of inadequate toothbrushing were found among male, Indian, had ever drug use, had three or more lack of protective factors and had inadequate hand hygiene practices. Adolescents who had inadequate toothbrushing were significantly higher odd among males, Indian ethnic, ever drug use, inadequate hand hygiene practices and adolescents who had three or more lack of peer and parental/guardian support.

**Conclusion:**

Approximately 1 out of 10 adolescents had inadequate toothbrushing practices with several factors associated, such as male gender, Indian ethnicity, inadequate hand hygiene, ever drug use and lack of protective factors are identified to be associated. By emphasizing the significance of frequent brushing, we can encourage positive changes and reduce the burden of preventable dental problems on adolescents.

## Background

Good oral health can be defined as a functional, structural, aesthetic, physiologic, and psychosocial state of well-being and is essential to an individual’s oral health and quality of life [1, 2]. Oral health care and good oral hygiene practices are important across all age groups. Since permanent teeth are already erupted during adolescence, oral health among adolescents is a strong predictor of adult oral health status.

Toothbrushing is one of the oral hygiene practices that has usually been highlighted and considered to be a good indicator in evaluating individual oral health care. Furthermore, toothbrushing can also be considered a fundamental self-care behaviour for the maintenance of oral health. Twice a day toothbrushing is considered a social norm in maintaining oral health through effective plaque control [3, 4]. As such, inadequate toothbrushing define as brushing teeth less than two times per day [5, 6]. Previous studies show the prevalence of inadequate toothbrushing among school adolescent (13–17 years old) in six Southeast Asian countries varies from 10.8% in Indonesia to 36.4% in Bangladesh [5].

Few studies have stated that inadequate toothbrushing is associated with the development of dental caries and periodontal disease [5, 7]. Burden of oral diseases varies among school children (11 to 12 years old), with caries prevalence of 26.3% in India [8], 33.3% in Malaysia [9] to 39.9% in China and caries severity with mean Decayed, Missing, and Filled Teeth (DMFT) of 0.17 among Malaysian school children to 3.29 among Brazilian school children [10]. Studies have shown that many factors are associated with inadequate toothbrushing practices among adolescents, such as male gender [5, 11, 12], health risk behaviours such as smoking [6, 11, 12], alcohol and drug use (such as cannabis) [13], inadequate exercise [6, 12], inadequate fruit/ vegetable intake [5, 13], psychological distress [5, 13], lack of peer and parental/guardian support [5, 13] and obesity [12]. These findings may indicate risky lifestyle behaviour that need to be highlighted when associating inadequate toothbrushing among adolescents.

In Malaysia, an adolescent health survey was conducted as a nationwide survey with the aim of assessing the prevalence of health risk behaviours and protective factors among adolescent students (13 to 17 years old) attending school, in conjunction with the Global School Health Survey (GSHS). Therefore, it is crucial for in-depth analysis to be conducted especially in exploring toothbrushing behaviour among adolescents, with relevant association with health risk behaviour and protective factors. In-depth analysis can further investigate the issue in detail and uncover the hidden patterns relationships between the inadequate toothbrushing with relevant factors. Thus, this paper will explore the prevalence of inadequate toothbrushing among 13- to 17-year-old in school adolescents in Malaysia as well as the association with relevant factors such as sociodemographic factors, health risk behaviours as well as factors associated.

## Methods

### Adolescent survey

This study was part of the GSHS 2017, which was a national cross-sectional survey designed to study adolescent health and risky lifestyle behaviour among in-school teenagers, aged between 13 to 17 years in Malaysia. The survey utilized a two-stage stratified sampling design to ensure the representativeness of school adolescents. The first stage of sampling was the selection of schools to represent each state and federal territory (16 states/federal territories) in Malaysia and the second stage was the selection of the class in the schools that were selected. Schools were randomly selected using probability sampling, proportionate by school enrolment size while the classes in school were selected using systematic random sampling. A total of 212 schools were selected with a range of 4 to 10 classes to meet the requirement sample for each state and federal territory. Upon selection, all students in the class were eligible to participate in the survey. No exclusion criteria were applied as the survey include all the students selected in the class. A total of 30,832 eligible students were recruited based on school registration and 27,497 students agreed to participate the survey with the consent from the parents, for a response rate of 89.2%. However, this study only includes data from 25,284 students due to incomplete data on the studied variables due to the students did not answer the modules. More information about this section can be obtained from the GSHS 2017 report [14].

### Survey instrument

Validated self-administered bilingual (Malay and English) questionnaires adopted from the Malaysia GSHS 2012 were used to capture adolescent health and risky lifestyle behaviour. The instruments used computer-scan-able answer sheets, which were also known as optical mark reader (OMR) sheets, to capture all the answers given by the respondents. The questionnaires comprised 77 questions that addressed topics related with adolescent health and risky lifestyles [14]. For this study, several topics were chosen to be included: substance use (alcohol, drug, smoking cigarettes), eating patterns, hygiene (inclusive of oral and hand hygiene), mental health status, peer and parental/guardian support, truancy, physical activity, and body mass index (BMI). In ensuring the quality of the data collection, a pilot study was conducted on 6th February 2017 at a few selected schools in state of Selangor. Data collection was conducted from 26th March until 3rd May 2017. Prior to data collection, a training course was held on 19th March 2017 for data collectors in Peninsular and East Malaysia.

### Hygiene

The module comprised two hygiene components in adolescent health; oral hygiene and hand hygiene. For the oral hygiene component, respondents were asked “During the past 30 days, how many times per day did you usually clean or brush your teeth?” with the response given as frequency numbers of brushing teeth per day. Brushing teeth less than 2 times and not cleaning or brushing for the past 30 days were categorized as inadequate toothbrushing practices, while others were categorized as adequate toothbrushing practices [6, 14].

In addition, there were two questions related to the oral hygiene component. The respondents were asked “Do you use dental floss to clean your teeth?” with the response either “yes” or “no”. Another question was “When was the last time you saw a dentist or dental nurse for a check-up, teeth cleaning, or other dental treatment?” with the response given as follows: a = during the past 12 months, b = between 12 and 24 months ago, c = more than 24 months ago, d = never, and e = I do not know. Respondents who chose during the past 12 months were considered routine attenders, while others were nonroutine attenders [14, 15].

The hand hygiene component comprised three questions related to daily hand washing practices. Respondents were asked for the past 30 days how often they practice the following behaviour: use of soap when washing their hands, wash hands before eating and wash hands after using the toilet. The responses given for each question were as follows: a = never, b = rarely, c = sometimes, d = most of the time, and e = always. Respondents who chose “most of the time” and “always” were considered had good practice, while others were considered poor practice. In this study, poor hand hygiene practice was considered if the respondents had at least one poor practice in any of the three questions given [14].

### Substance use

For smoking cigarettes, respondents were asked “During the past 30 days, on how many days did you smoke cigarettes?”. In addition, the respondents were asked if they smoked e-cigarettes or vape for the past 30 days, with the same question structure as smoking cigarettes. For alcohol drinker status, the respondents were asked “During the past 30 days, on how many days did you have at least one drink containing alcohol?”. Both smoking and alcohol drinker status had the same answer selection: a = 0 days, b = 1 or 2 days, c = 3 to 5 days, d = 6 to 9 days, e = 10 to 19 days, f = 20 to 29 days, and g = all 30 days. Respondents were considered current users (for each substance) if they smoked or drank at least 1 out of 30 days. For drug use, the respondents were asked “During your life, how many times have you used drugs?” with the response as follows: a = 0 times, b = 1 or 2 times, c = 3 or 9 times, d = 10 to 19 times, and e = 20 or more times. Respondents were considered to have ever used drug if they had a history (at least 1 time) in their life, regardless of drug type [14].

### Eating pattern

Respondents were asked four eating patterns which were direct questions regarding their food/drink intake or serving. Respondents were asked “During the past 7 days, how many days did you eat food from fast food restaurants, such as McDonalds, KFC and Pizza Hut?” with the response option in days. Respondents were considered had frequent fast-food intake if they took 3 days or more. In addition, with the fast-food intake, the respondents were also asked “During the past 30 days, how many times per day did you usually drink carbonated soft drinks such as Coca Cola, Sprite and Pepsi?” with the response also in days. Respondents were considered carbonated drinkers if they consumed at least 1 time per day during the past 30 days. For fruit or vegetable serving intake, the respondents were asked how many times per day they had eaten fruits or vegetables for the past 30 days. Both questions had the same response in days. Respondents who reported that they consumed fruits (or vegetables) 1 times per day or none were considered had inadequate consumption patterns [14].

### Mental health status

A standardized, validated and translated bilingual Depression Anxiety and Stress Scale (DASS) 21 was used to determine the mental health status of the respondents [16]. The questionnaire comprises 21 questions with four answer selections as follows: a = did not apply to me at all, b = applied to me to some degree or some of the time, c = applied to me to a considerable degree or a good part of time, and d = applied to me very much, or most of the time. The scoring and grouping of the mental health status (depression, anxiety, stress) followed the standard procedure of using the DASS 21 [14, 16].

### Peer and parental/guardian support

Peer and parental/guardian support were assessed with five items on peer support at school, parental or guardian supervision, connectedness, bonding, and respect for privacy of the respondents. Peer support at school was assessed with the question “During the past 30 days, how often were most of the students in your school kind and helpful?”, parental or guardian supervision “During the past 30 days, how often did your parents or guardians check to see if your homework was done?”, parental or guardian connectedness “During the past 30 days, how often did your parents or guardians try to understand your problems and worries?”, parental or guardian bonding “During the past 30 days, how often did your parents or guardians truly know what you were doing with your free time?” and parental or guardian respect privacy “During the past 30 days, how often did you parents or guardians go through your things without your approval?”. All the items had the same response option as follows: a = never, b = rarely, c = sometimes, d = most of the time, and e = always. For peer support at school, parental or guardian supervision, connectedness and bonding items, respondents were considered had peer and support factors if they chose always and most of the time in the response selection. However, for the respect for privacy item, respondents were only considered had peer and support factors if they selected never or rarely in the response selection [14].

### Truancy

Respondents were asked “During the past 30 days, on how many days did you miss classes or school without permission?” with response options in days. Respondents were considered had truancy if they missed class or school at least 1 day in the past 30 days [14].

### Physical activity

Respondent’s physical activity was assessed based on the self-reported answer on the OMR sheet. Respondents were asked “During the past 7 days, on how many days were you physically active for a total of at least 60 minutes per day?” with the response option in days. Respondents were considered active for at least 60 minutes per day (sum of all the time spent in any kind physical activities), with a minimum of 5 days per week [17]. Sedentary behaviour was also assessed by asking the respondents “How much time do you spend during a typical or usual day sitting and watching television, playing computer games, talking with friends, or doing other sitting activities?” with response in hours per day. Respondents were considered had sedentary behaviour if they spent at least 3 hours in sitting activities [14, 17].

### Body mass index (BMI)

Height and body weight were assessed by using a portable measuring scale, in the presence of data collectors. The international age-gender specific child BMI cut off points were used in this study to define the underweight, normal, and overweight/obesity status of the respondents [17, 18]. The interpretation of cut-offs as follow: Overweight: >+1SD (equivalent to BMI 25 kg/m2 at 19 years), Obesity: >+2SD (equivalent to BMI 30 kg/m2 at 19 years), Thinness: <-2SD, Severe thinness: <-3SD, normal: 0. The interpretation was based on z-scores chart and well-trained nurses evaluated the age-gender specific child BMI cut off points for each student [14, 17, 18].

### Ethics approval and consent to participate

Ethical approval for this study was obtained from the Medical Research and Ethics Committee (MREC), Ministry of Health Malaysia and registered with the National Medical Research Register (NMRR-16-698-30042). The survey follows the guidelines and regulations provided by the Ministry of Health Malaysia to abide by the ethics in this study. All the participants were given a written consent form prior to data collection. All the participants and legal guardians / parents were briefly informed by the written consent form on the study conducted before given their consent to participated their children. Informed consent was then obtained from all the participants and legal guardians/parents for the study before the data collection start.

### Data analysis

Data analysis was performed using IBM SPSS for Windows, Version 26.0. The prevalence of inadequate toothbrushing was determined using complex sampling analysis with a 95% confidence interval applied, and a weighting factor was used to adjust for non-response students and for varying probabilities of selection. Factors associated with inadequate toothbrushing practice were determined at both univariate and multivariable levels using simple logistic regression and multiple logistic regression respectively. Independent variables such as sociodemographic and factors associated that had been included in this study were used in the model. The outcome was a binary variable coded as “0” for adequate toothbrushing practice and “1” for inadequate toothbrushing practice among in-school adolescents. For crude odd ratio, the p-value less than 0.25 were determine as acceptable criteria to be included in adjusted odd ratio analysis. The goodness of fit of the model was determined using the Hosmer-Lemeshow goodness-of-fit test with a non-significant p value (>0.05). Interactions between variables and multicollinearity must show no significant interaction result, and the classification table value with receiver operating characteristics (ROC) curve value were also determined, with the value at least 0.70. The final model was presented with an adjusted odds ratio (aOR) and 95% confidence interval (95% CI), with the level of significance set at p-value of less than 0.05 to ensure the quality of the model.

## Results

### Prevalence of inadequate toothbrushing

Overall, approximately 12.7% (95% CI: 11.8–13.6) of in-school adolescents had inadequate toothbrushing practices during the past 30 days in Malaysia. In sociodemographic, higher prevalence was seen in male adolescents (17.9%, 95% CI: 16.7–19.3), Chinese (21.7%, 95% CI: 19.7–23.9), Indian (21.4%, 95% CI: 17.2–26.3) and adolescents who had their parents divorced/widow(er)/separated (15.3%, 95% CI: 13.6–17.2), respectively (Table 1).

**Table 1:** Prevalence of inadequate toothbrushing practice, risky and unhealthy factors among in-school adolescents.

Variables	Count	Estimated population	Prevalence (%)	95% CI	
				Lower	Upper
**Overall**	2,984	248,788	12.7	11.8	13.6
**State & Federal**					
Johor	176	30,124	11.6	9.7	13.7
Kedah	173	16,098	11.4	9.2	14.0
Kelantan	184	14,093	13.1	10.6	16.0
Melaka	189	6,231	9.9	7.9	12.2
Negeri Sembilan	184	10,241	12.5	10.0	15.7
Pahang	154	9,429	10.1	7.5	13.4
Pulau Pinang	201	13,796	13.1	10.1	16.9
Perak	261	27,117	16.2	12.9	20.2
Perlis	137	2,399	9.8	7.6	12.5
Selangor	236	54,221	15.2	12.1	18.8
Terengganu	140	8,534	9.6	7.8	11.8
Sabah	136	16,242	9.7	6.8	13.6
Sarawak	189	20,182	11.0	9.2	13.2
W.P. Kuala Lumpur (Federal)	248	18,431	17.2	14.1	20.9
W.P. Labuan (Federal)	146	499	9.9	7.8	12.6
W.P. Putrajaya (Federal)	230	1,151	14.1	11.8	16.8
**Age**					
≤ 13 years old	639	53,425	13.5	11.4	15.9
14–15 years old	1,268	99,081	12.5	11.4	13.7
≥ 16 years old	1,077	96,283	12.4	11.2	13.8
**Residence**					
Urban	1,852	152,878	13.5	12.3	14.9
Rural	1,132	95,910	11.5	10.3	12.9
**Gender**					
Male	2,059	173,151	17.9	16.7	19.3
Female	925	75,637	7.6	6.6	8.7
**Ethnicities**					
Malays	1,724	123,691	10.0	9.2	10.7
Chinese	755	73,061	21.7	19.7	23.9
Indian	238	28,594	21.4	17.2	26.3
East Malaysia (Sabah & Sarawak)	210	19,158	8.9	6.6	11.8
Others	57	4,283	12.9	9.2	17.7
**Parents Marital Status**					
Married	2,345	196,102	12.1	11.3	13.0
Divorced/Widow(er)/Separated	639	52,686	15.3	13.6	17.2
**Eating Pattern**					
**Fruits Serving intake**					
Adequate	899	75,166	8.2	7.4	9.0
Inadequate	2,085	173,622	16.6	15.5	17.8
**Vegetables Serving intake**					
Adequate	766	67,018	9.5	8.5	10.7
Inadequate	2,218	181,770	14.5	13.4	15.6
**Soft drinks at least once daily**					
Do not consume	1,955	159,753	12.8	11.8	13.8
Consume	1,029	89,035	12.5	11.3	13.9
**Fast foods intake at least 3 times/week**					
Do not consume	371	30,851	14.7	12.2	17.7
Consume	2,613	217,937	12.4	11.6	13.3
**BMI**					
Normal	1,814	152,595	12.0	11.1	12.9
Underweight	206	16,891	13.3	11.3	15.5
Overweight/Obese	964	79,303	14.1	12.8	15.5
**Physical Activity (PA)**					
**PA Status**					
Active	310	25,805	11.4	9.9	13.1
Inactive	2,674	222,983	12.9	11.9	13.9
**Sedentary Status**					
Yes	1,633	135,218	13.6	12.5	14.8
No	1,351	113,570	11.7	10.7	12.8
**Substance Use**					
**Smoker Status**					
Yes	648	56,841	21.9	18.8	25.2
No	2,336	191,947	11.3	10.5	12.1
**E-smoker Status**					
Yes	443	38,473	20.9	17.7	24.5
No	2,541	210,315	11.8	11.0	12.7
**Alcohol Drinker**					
Yes	523	49,731	25.6	22.4	29.0
No	2,461	199,057	11.3	10.5	12.1
**Ever Drug Use**					
Yes	311	29,034	38.3	32.4	44.5
No	2,673	219,754	11.7	10.9	12.5
**Truancy**					
Yes	1,010	87,606	15.5	14.1	17.1
No	1,974	161,182	11.5	10.7	12.5
**Psychological Status**					
Zero Status	1,552	129,078	11.5	10.6	12.4
At least 1 status	729	59,210	11.9	10.5	13.4
≥ 2 statuses	703	60,500	17.7	15.2	20.5
**Peer and parental/guardian support**					
Zero Lack of Peer and Support	31	2,358	4.9	3.3	7.4
1 Lack of Peer and Support	118	10,080	7.0	5.5	9.0
2 Lack of Peer and Support	328	27,032	9.5	8.0	11.2
≥ 3 Lack of Peer and Support	2,507	209,318	14.1	13.1	15.1
**Hygiene Component**					
**Hand Hygiene Practices**					
Good	1,022	84,828	8.0	7.3	8.7
Poor	1,962	163,960	18.3	16.9	19.8
**Dental Floss Usage**					
Yes	582	53,540	14.4	12.6	16.3
No	2,402	195,248	12.3	11.5	13.2
**Dental Check up**					
Routine attenders	1,060	79,583	9.9	9.0	11.0
Non-routine attenders	1,924	169,205	14.6	13.5	15.7

For risky and unhealthy behaviour, higher prevalence was seen in adolescents who had inadequate fruit servings (16.6%, 95% CI: 15.5–17.8), inadequate vegetable servings (14.5%, 95% CI: 13.4–15.6), current smokers (21.9%, 95% CI: 18.8–25.2), current e-smokers (20.9%, 95% CI: 17.7–24.5), current alcohol drinkers (25.6%, 95% CI: 22.4–29.0) and ever drug users (38.3%, 95% CI: 32.4–44.5), respectively (Table 1). In terms of social, higher prevalence was seen in adolescents who reported had three or more lack of peer and parental/guardian support (14.1%, 95% CI: 13.1–15.1), truancy (15.5%, 95% CI: 14.1–17.1) had two or more mental issues (anxiety, stress, depression) (17.7%, 95% CI: 15.2–20.5), respectively (Table 1).

For the hygiene component, adolescents who reported poor hand hygiene practices also had a higher prevalence of inadequate toothbrushing practices (18.3%, 95% CI: 16.9–19.8) than good hand hygiene practices. In addition, adolescents who reported as non-routine attenders (14.6%, 95% CI: 13.5–15.7) showed a higher prevalence than those routine attenders, respectively (Table 1).

By fitting all the sociodemographic and relevant factors, multiple logistic regressions showed that few sociodemographic and health behaviours were associated with inadequate toothbrushing practices. In sociodemographic, higher odds of inadequate toothbrushing were found significantly associated in male adolescents (aOR 2.46, 95% CI: 2.26–2.69) and among Indian adolescents (aOR 2.14, 95% CI: 1.82–2.52). For substance use, adolescents who had ever used drug also showed higher probability of inadequate toothbrushing practices, with a significantly associated of aOR of 2.53 (95% CI: 2.13, 3.00). In addition, adolescents who had three or more lack of peer and parental/guardian support (aOR 2.17, 95% CI: 1.49–3.17) and poor hand hygiene practices (aOR 2.01, 95% CI: 1.84–2.19) had significantly higher probability associated of inadequate toothbrushing practices. Other variables such as Chinese adolescents, parents’ marital status (divorced/widow(er)/separated), inadequate fruit intake, inadequate vegetable intake, overweight/obese BMI, inactive physical activity, sedentary status, current smoker, had psychological statuses and non-routine attenders also showed higher odds of inadequate toothbrushing practice. However, the value was considered low compared to other variables highlighted. Other variables were found not significant in this study (Table 2).

**Table 2:** Association of inadequate toothbrushing practice with sociodemographic and risky and unhealthy factors among in-school adolescents.

Variables	Simple Logistic Regression (SLR)				Multiple Logistic regression (MLR)			
	cOR	95% CI		p-value	aOR	95% CI		p-value
		Lower	Upper			Lower	Upper	
**Age**								
≤ 13 years old	1	-	-	-	1	-	-	-
14–15 years old	0.96	0.87	1.06	0.45	0.96	0.86	1.07	0.432
≥ 16 years old	0.87	0.78	0.96	0.006	0.87	0.78	0.97	0.056
**Strata**								
Urban	1	-	-	-	1	-	-	-
Rural	0.86	0.79	0.93	<0.001	1.01	0.92	1.10	0.916
**Gender**								
Male ^**b**^	2.77	2.55	3.01	<0.001	**2.46**	**2.26**	**2.69**	**<0.001**
Female	1	-	-	-	1	-	-	-
**Ethnicities**								
Malays	1	-	-	-	1	-	-	-
Chinese	2.21	2.01	2.43	<0.001	**1.79**	**1.62**	**1.98**	**<0.001**
Indian ^**b**^	2.03	1.75	2.36	<0.001	**2.14**	**1.82**	**2.52**	**<0.001**
East Malaysia (Sabah & Sarawak)	0.87	0.74	1.01	0.058	0.81	0.69	0.95	0.051
Others	1.18	0.89	1.56	0.282	1.09	0.81	1.46	0.37
**Parents Marital Status**								
Married	1	-	-	-	1	-	-	-
Divorced/Widow(er)/Separated	1.36	1.24	1.49	<0.001	**1.13**	**1.02**	**1.25**	**0.016**
**Eating Pattern**								
Inadequate fruits intake	2.21	2.04	2.40	<0.001	**1.78**	**1.63**	**1.94**	**<0.001**
Inadequate vegetables intake	1.61	1.48	1.76	<0.001	**1.31**	**1.20**	**1.44**	**<0.001**
Soft drinks at least once daily	0.97	0.90	1.05	0.475	-	-	-	-
Fast foods intake at least 3 times/week	1.20	1.07	1.35	0.002	1.11	0.97	1.26	0.123
**BMI**								
Normal	1	-	-	-	1	-	-	-
Underweight	1.24	1.07	1.45	0.006	1.00	0.85	1.12	0.996
Overweight/Obese	1.21	1.11	1.32	<0.001	**1.25**	**1.14**	**1.36**	**<0.001**
**Physical Activity**								
Inactive	1.24	1.10	1.41	0.001	**1.18**	**1.04**	**1.35**	**0.012**
Sedentary	1.20	1.11	1.30	<0.001	**1.13**	**1.04**	**1.22**	**0.004**
**Substance Use**								
Current Smoker	2.18	1.98	2.40	<0.001	**1.28**	**1.14**	**1.43**	**<0.001**
Current E-smoker	2.00	1.79	2.23	<0.001	0.91	0.79	1.06	0.241
Current Alcohol Drinker	2.63	2.37	2.93	<0.001	1.10	0.96	1.27	0.170
Ever Drug Use ^**b**^	4.86	4.20	5.63	<0.001	**2.53**	**2.13**	**3.00**	**<0.001**
**Truancy**	1.34	1.23	1.45	<0.001	1.06	0.97	1.16	0.238
**Psychological Status**								
Zero Status	1	-	-	-	1	-	-	-
At least 1 status	1.08	0.98	1.19	0.111	**1.14**	**1.03**	**1.26**	**0.010**
≥ 2 statuses	1.64	1.49	1.81	<0.001	**1.23**	**1.10**	**1.37**	**<0.001**
**Peer and parental/guardian support**								
Zero Lack of Peer and Support	1	-	-	-	1	-	-	-
1 Lack of Peer and Support	1.33	0.89	1.99	0.171	1.42	0.93	2.17	0.101
2 Lack of Peer and Support	1.87	1.28	2.73	0.001	**1.68**	**1.13**	**2.49**	**0.010**
≥ 3 Lack of Peer and Support	3.07	2.14	4.42	<0.001	**2.17**	**1.49**	**3.17**	**<0.001**
**Hygiene Component**								
Poor Hand Hygiene Practices ^**b**^	2.65	2.45	2.88	<0.001	**2.01**	**1.84**	**2.19**	**<0.001**
Do not use dental floss	0.99	0.85	1.03	0.162	1.00	0.90	1.11	0.99
Non-routine attenders	1.44	1.33	1.56	<0.001	**1.21**	**1.11**	**1.31**	**<0.001**

^a^ All variables were adjusted for inadequate toothbrushing practice and backwards LR multiple logistic regression was applied. Multicollinearity and interaction were checked and not found. Hosmer-Lameshow test P value = 0.193. Classification table: 88.3. ROC: 0.745 (95% CI: 0.735, 0.754; p-value: <0.001)

^**b**^ Higher odd was found

## Discussion

Toothbrushing practice is one of the essential habits in achieving good oral health status. Effective removal of dental biofilm through toothbrushing and the use of fluoridated toothpaste is important in preventing the formation of dental caries [3, 19]. In this study, the prevalence of inadequate toothbrushing practice among adolescents was 12.7%, which is higher in Indonesia but much lower than in the Philippines, Thailand, Timor-Leste [5], Finland [11], Korean adolescents [6] and African countries [13]. Inadequate toothbrushing is considered factor associated for periodontitis [7, 20] and dental caries [3, 4]. Carious lesion occurred as time progress with inadequate toothbrushing during younger age. The lesion can lead severe damage to the teeth with tooth loss appeared in the final stage. Thus, it would suggest frequent toothbrushing can prevent the tooth disease, as per findings from previous studies [3, 4]. Even though the prevalence among adolescent is low, precautions are needed to prevent the poor practice to become nuisance in adult stage where other oral disease tend to occur. A previous oral health survey among adults in Malaysia in 2010 showed high oral disease burden with a dental caries prevalence of 88.9% (95% CI: 88.1, 89.6), and 94.0% (95% CI: 93.2, 94.8) presented with periodontal conditions [21]. Without proper intervention, dental caries will be a continuous process, which will lead to a high disease burden. Thus, it is crucial for preventive measures, such as frequent toothbrushing to reduce the oral health burden in adults as age continues.

In this study, male gender showed a higher prevalence and odds for inadequate toothbrushing practice. Gender differences in the adoption of preventive oral health care behaviours have been shown in other studies [22–27]. Female gender was reported to be more aware of the prevention of oral diseases [22], frequent toothbrushing [23–27], use of interdental floss and preventive dental check-ups [25] than their male counterparts. Female gender takes greater care of their oral hygiene regardless of age. Therefore, it is crucial to inculcate the importance of oral hygiene care, especially toothbrushing frequency. By ethnicity, Indians showed higher results in both prevalence and odds for inadequate toothbrushing compared to other ethnicities. Several factors may be associated with inadequate toothbrushing practices such as socio-cultural aspects and the perceived importance of good oral hygiene care. Other study also highlighted the negligence and lack of attitude toward oral hygiene among Indian ethnic has also a negative impact on the general hygiene [28]. With the negligence of the inadequate toothbrushing, periodontal diseases are among the most prevalent in the ethnic group [28]. Focus group discussions (FGD) or qualitative studies should be conducted among adolescents in Malaysia to better understand their inadequate behaviours.

Poor hand hygiene among adolescents is also associated with inadequate toothbrushing practices in this study. Similar findings were also observed among Iranian adolescents where there was an association between oral hygiene and general hygiene [23, 28, 29]. Poor hand hygiene and inadequate toothbrushing practices among adolescents can cause multiple disease burdens, and these poor habits may continue during adult life if no intervention is taken. Other study stated hand and oral hygiene practice are recommended as the most cost-effective way to reduce the spread of infectious diseases, as well as for reducing burden towards hygiene related diseases [29]. Study showed that not always or inadequate hand washing practice is associated with poor subjective oral health status, experience of oral disease symptoms, low frequency of brushing teeth, and non-use of oral supplements [29]. Therefore, well practice hand hygiene and oral hygiene practice in adolescence can have a lasting impact on health during growth and adulthood, reducing the burden of disease related with hygiene.

Another study suggested that attitudes, knowledge, beliefs, and self-efficacy are some of the measures that are thought to be on the causal pathway for behavioural changes [30]. Thus, adolescents are needed to also be exposed to the importance of maintaining good hand hygiene as well as oral hygiene. Good hand washing is important, especially for children and adolescents, as these groups are the most susceptible to infections gained from unwashed hands [30]. Other study also reported the lack of financial support, low parental knowledge, and unhealthy hygiene practice in adolescents’ families can contribute to inadequate oral hygiene practice among adolescents [29]. Therefore, education programs to spread awareness regarding the importance of preventive personal hygiene such as adequate hygiene practice should be recommended for parents as well as adolescents [29].

This study also found that a lack of peer and parental/guardian support and substance drug use were also associated with inadequate toothbrushing practices, which is consistent with other studies [5, 13, 27]. By practicing good oral hygiene, the prevalence of common oral disease can be prevented through frequent brushing practices. Inadequate oral hygiene and toothache had a significant association with inadequate school performance and school absenteeism [31]. Interventions to facilitate health-related behaviours should be made and geared to the most important risk factors related to unhealthy behaviours.

There were few limitations in this study. The survey was under GSHS, which only enrolled adolescents at the school. School-going adolescents may not be representative of all adolescents in a country, as the occurrence of frequent toothbrushing practice may differ between the two groups. The data obtained are more self-reported or perceived by the respondents which may lead to some biases. Furthermore, the data collection used the recall method. However, it should be reinforced that this study had great strength in using a nationally representative adolescent sample. The sampling units were based on the population and original school frame from the Ministry of Education Malaysia and the survey sample weights were adjusted to represent the adolescent population. In addition, the adolescents completed the questionnaires anonymously. This indicates that the results from this study are reliable and representative.

## Conclusion

In summary, approximately 1 in 10 adolescents had inadequate toothbrushing practices. Adolescents who were male, Indian, had ever used drug, had three or more lack of peer and parental/guardian support, and poor hand hygiene practices had a higher prevalence of inadequate toothbrushing, with in-depth analysis showing an association of the factors mentioned with inadequate behaviour. Understanding the root of cause for inadequate toothbrushing is essential in developing targeted interventions and educational programs to encourage adolescents to adopt healthier oral care practices. Emphasizing the significance of frequent brushing, we can encourage positive changes and reduce the burden of preventable dental problems on adolescents.
